# Association between visceral adiposity index and delayed union/nonunion after tibial or femoral shaft fractures: a single-center retrospective cohort study

**DOI:** 10.1186/s12891-026-10074-x

**Published:** 2026-06-20

**Authors:** Xiaohai Luo, Ning Wu, Zhaofu Wang, Ermei Lu, Xiaoyu Zhang

**Affiliations:** 1 Orthopedics Department, People’s Hospital of Ningxia Hui Autonomous Region, 301 Zhengyuan North Street, Yinchuan, Ningxia, 750002 China; 2 Ningxia Integrated Chinese and Western Medicine Hospital, Yinchuan, 750021 China; 3 The First Clinical Medical College, Lanzhou University, Lanzhou, 730000 China

**Keywords:** Visceral adiposity index, Delayed union, Nonunion, Tibial shaft fracture, Femoral shaft fracture, Machine learning

## Abstract

**Background:**

Delayed union and nonunion remain clinically important complications after tibial and femoral shaft fractures. Although traditional risk factors such as smoking and diabetes have been widely investigated, the association between visceral adiposity-related metabolic dysfunction and impaired fracture healing remains unclear. This study aimed to examine the association between the visceral adiposity index (VAI) and delayed union/nonunion after tibial or femoral shaft fractures and to explore whether VAI could improve internally validated prediction models.

**Methods:**

We conducted a single-center retrospective cohort study of 485 adults who underwent intramedullary fixation for isolated tibial or femoral shaft fractures between January 2022 and June 2025. VAI was calculated using sex-specific equations incorporating waist circumference, body mass index, triglycerides, and high-density lipoprotein cholesterol. The primary endpoint was delayed union/nonunion at 6 months after surgery. The association between VAI and delayed union/nonunion was assessed using multivariable logistic regression and restricted cubic spline analysis. Discriminative performance was compared using receiver operating characteristic analysis, and exploratory machine learning models were evaluated using internal validation.

**Results:**

Delayed union/nonunion occurred in 60/485 patients (12.4%). The median VAI was higher in patients with delayed union/nonunion than in those who achieved fracture union [3.1 (IQR, 2.2–4.5) vs. 1.9 (IQR, 1.3–2.8), *P* < 0.001]. After full adjustment for demographic, lifestyle, metabolic, and fracture-related covariates, each 1-unit increase in VAI was associated with higher odds of delayed union/nonunion (OR 1.61, 95% CI 1.31–1.98; *P* < 0.001). Restricted cubic spline analysis suggested an approximately linear association, without significant evidence of nonlinearity. VAI showed higher discrimination than body mass index and waist circumference (AUC 0.785 vs. 0.655 and 0.712, respectively). In exploratory internal validation, L1/Elastic Net-regularized logistic regression showed the highest AUC among the evaluated machine learning models (AUC 0.914, 95% CI 0.864–0.963).

**Conclusion:**

In this single-center retrospective cohort, higher VAI was associated with an increased risk of delayed union/nonunion after tibial or femoral shaft fractures treated with intramedullary fixation. VAI showed better discrimination than conventional anthropometric measures in internal analyses and may provide additional information for early risk stratification. However, these findings do not establish causality, and external prospective validation is required before clinical implementation.

Long bone shaft fractures of the tibia and femur are among the most frequently encountered injuries in adult orthopaedic trauma and are often accompanied by substantial functional limitations during recovery. Despite advances in internal fixation techniques and perioperative management, delayed union and nonunion remain clinically significant complications, with reported incidences ranging from approximately 5% to 15% depending on fracture location and patient characteristics [1, 2]. Failure of timely bone healing often necessitates additional surgical interventions, prolongs rehabilitation, and contributes to increased healthcare costs and long-term functional impairment [3, 4]. Consequently, identifying patients at elevated risk before biological failure becomes radiographically evident remains a major clinical priority.

Fracture healing is a tightly regulated regenerative process involving coordinated phases of inflammation, angiogenesis, cellular recruitment, and bone remodeling [5–7]. Beyond local mechanical stability and fracture severity, systemic host-related factors substantially influence the biological environment of repair. Established risk factors for nonunion include advanced age, smoking, and diabetes mellitus [8–11]. However, these variables capture isolated clinical conditions and may not adequately reflect the broader metabolic milieu of the patient. Increasing evidence suggests that obesity and metabolic dysfunction interact with bone metabolism in complex ways. Emerging data indicate that metabolic disturbances and excess adiposity influence skeletal biology through immunometabolic pathways. In this context, adipose tissue functions not merely as an energy reservoir but as an endocrine organ capable of releasing cytokines and adipokines that modulate osteoblast–osteoclast balance and inflammatory signaling within the bone–immune axis [12–14].

The visceral adiposity index (VAI) integrates waist circumference (WC), BMI, and circulating lipid parameters to approximate visceral fat dysfunction and cardiometabolic risk [15, 16]. Previous studies have demonstrated associations between VAI and insulin resistance, metabolic syndrome, and chronic inflammatory states [14–16]. However, its potential relevance in the context of fracture healing has not been systematically investigated. In particular, clinical evidence linking visceral adiposity–related dysfunction to healing outcomes in long bone fractures remains limited.

Moreover, existing prediction models for fracture nonunion are largely based on radiographic assessments or postoperative variables [17–19], which may identify risk only after biological impairment has progressed. With the rapid development of predictive analytics and machine learning approaches, incorporating systemic metabolic markers into risk assessment frameworks may enhance discriminative performance and improve model interpretability. Nevertheless, data evaluating visceral adiposity–related indicators within fracture healing prediction models are currently lacking.

Therefore, the present study aimed to examine the association between VAI and delayed union/nonunion following long bone fractures, to characterize potential dose–response relationships, and to develop integrated predictive models combining metabolic and clinical variables. By approaching fracture healing from a systemic metabolic perspective, this study seeks to refine risk assessment strategies and provide additional insights into individualized management.

## Methods

### Study design and population

**Fig. 1:**
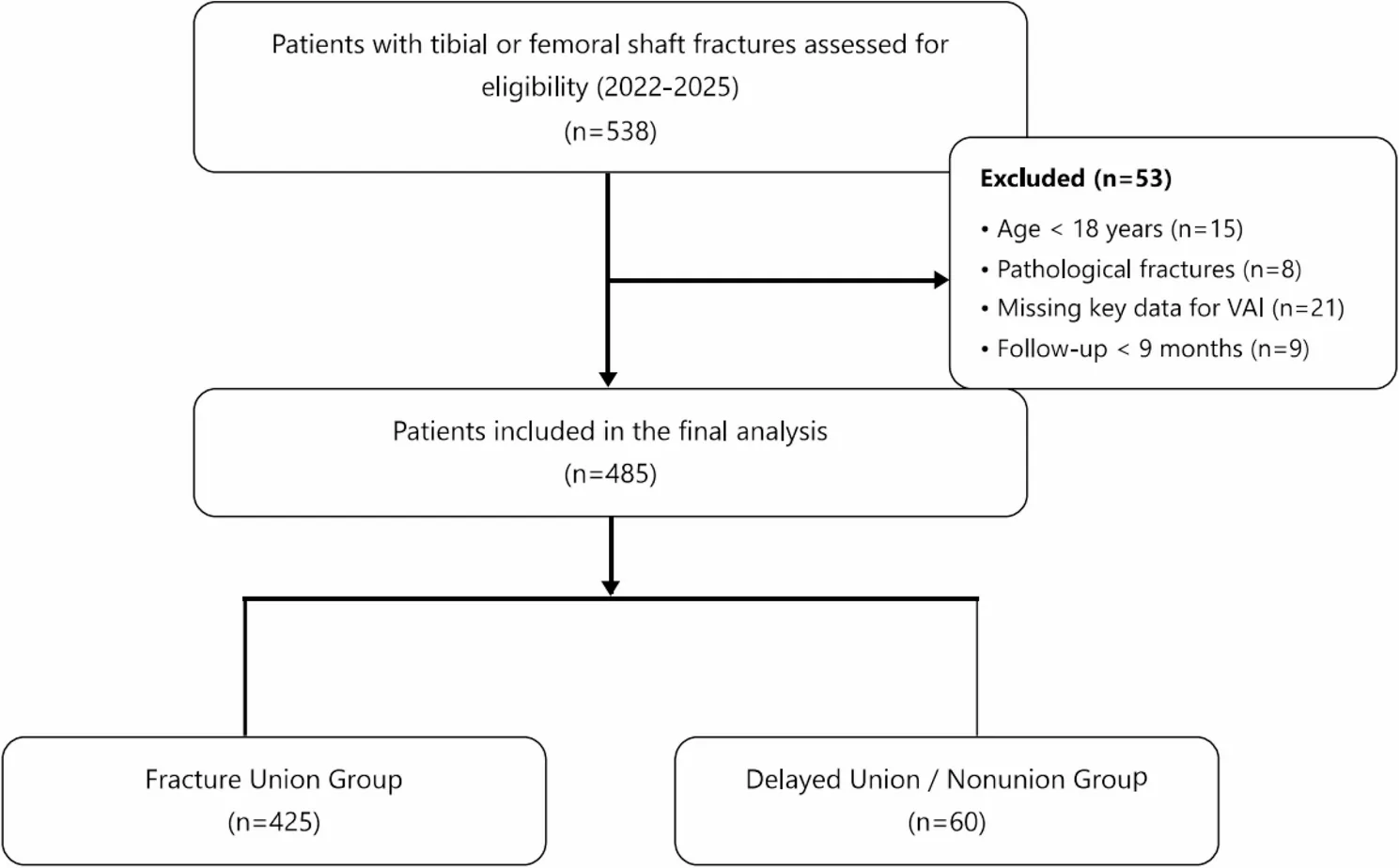
Flow diagram of the patient selection process

We conducted a single-center retrospective cohort study of consecutive adult patients who underwent intramedullary fixation for isolated tibial or femoral shaft fractures at the People’s Hospital of Ningxia Hui Autonomous Region between January 2022 and June 2025. Eligible cases were identified from the institutional electronic medical record system based on the index hospitalization for intramedullary fixation. The patient screening procedure is summarized in Fig. 1. The study was approved by the institutional ethics committee (Approval No. LL-050), and the requirement for informed consent was waived due to the retrospective nature of the analysis.

Inclusion criteria were as follows: (1) age ≥ 18 years; (2) radiologically confirmed isolated, closed tibial or femoral shaft fracture; (3) treatment with intramedullary nailing; and (4) available radiographic follow-up for assessment of fracture healing at 6 months.

Exclusion criteria: (1) open fractures or polytrauma; (2) pathological fractures (e.g., due to tumors or metabolic bone disease); (3) pre-existing severe systemic diseases known to affect bone metabolism; and (4) significant missing clinical, biochemical, or follow-up data.

### Data collection and variable definition

Baseline demographic, clinical, anthropometric, and laboratory data were extracted from hospital records. Demographic variables included age and sex. Clinical covariates comprised smoking status (current use or cessation within 1 year), diabetes mellitus, fracture location, and injury mechanism. Fracture complexity was categorized according to the AO/OTA classification system (Type A: simple; Type B: wedge; Type C: complex).

Anthropometric measurements were obtained at admission. Body mass index (BMI) was calculated as weight in kilograms divided by height in meters squared. Waist circumference (WC) was measured under fasting conditions. Fasting blood samples collected on the morning following admission were used to determine triglycerides (TG), high-density lipoprotein cholesterol (HDL-C), and albumin concentrations.

The VAI was computed using established sex-specific equations incorporating BMI, WC, TG, and HDL-C levels. VAI is a unitless index:

Males:

$$\begin{aligned} \mathrm{VAI}& = \left(\mathrm{WC} / \left(39.68+1.88 \times \mathrm{BMI}\right)\right) \\&\times \left(\mathrm{TG} / 1.03\right) \times \left(1.31 / \mathrm{HDL-C}\right) \end{aligned}$$

Females:

$$\begin{aligned} \mathrm{VAI} &= \left(\mathrm{WC} / \left(36.58+1.89 \times \mathrm{BMI}\right)\right) \\&\times \left(\mathrm{TG} / 0.81\right) \times \left(1.52 / \mathrm{HDL-C}\right) \end{aligned}$$

(WC in cm; TG and HDL-C in mmol/L)

### Outcome assessment

The primary endpoint was delayed union/nonunion at 6 months after surgery. Delayed union and nonunion were combined as a composite endpoint because both reflect impaired fracture healing and because the number of nonunion events was limited. Outcome assessment was primarily radiographic. Serial radiographs were independently reviewed by two senior orthopaedic surgeons. Discordant evaluations were adjudicated through consensus or consultation with a third reviewer. Delayed union/nonunion was defined as absence of cortical bridging and lack of progressive callus formation across three consecutive monthly radiographs, in the absence of infection. Patients not meeting these criteria were classified as having achieved fracture union.

### Statistical analysis

All analyses were conducted using R software (version 4.2.1 or later). Continuous variables were summarized according to distribution characteristics and compared using parametric or nonparametric tests as appropriate. Categorical data were analyzed using chi-square or Fisher’s exact testing. Patients with missing data on VAI components, key covariates, or radiographic healing outcomes were excluded before analysis. The final analysis was therefore performed using a complete-case approach, and no multiple imputation was performed.

The association between VAI and delayed union/nonunion was evaluated using logistic regression modeling. Sequential adjustment was performed: an unadjusted model; a model controlling for age and sex; and a fully adjusted model additionally incorporating smoking, diabetes mellitus, AO classification, and injury mechanism. VAI was modeled both continuously (per unit increase) and categorically by quartiles, with trend tests performed across ordered groups. Effect estimates are reported as odds ratios (ORs) with 95% confidence intervals (CIs).

To investigate potential nonlinearity, restricted cubic spline (RCS) functions were fitted within the fully adjusted framework. Interaction terms were introduced to assess sex-specific modification of the association.

The discriminative performance of VAI was compared with BMI and WC using receiver operating characteristic (ROC) analysis, and differences in the area under the curve (AUC) were tested using the DeLong method. Statistical significance was defined as a two-sided P value < 0.05.

### Exploratory machine learning model development and internal validation

To explore the potential value of VAI in risk prediction, we developed and internally validated ten supervised machine learning models.

### Data preprocessing and splitting

The entire dataset (*n* = 485) was randomly partitioned into a training set (70%, *n* = 340) and a validation set (30%, *n* = 145). Stratified sampling was used to maintain a consistent proportion of the outcome event (delayed union/nonunion) in both sets. In the training set, continuous features were standardized using Z-score normalization, and categorical features were transformed using one-hot encoding. To reduce the risk of data leakage, preprocessing steps, including Z-score normalization and one-hot encoding, were fitted within the training set and then applied to the validation set.

### Model training and selection

Ten models were developed on the training set: logistic regression with L1/Elastic Net regularization [LR(L1/EN)], gradient boosting machine (GBM), support vector machine with a linear kernel (SVM-L), random forest (RF), extreme gradient boosting (XGB), support vector machine with a radial basis function kernel (SVM-R), decision tree (DT), neural network (NN), naïve Bayes (NB), and k-nearest neighbors (KNN). A combination of grid search and 10-fold cross-validation was employed on the training set to identify the optimal hyperparameters for each model.

### Model performance evaluation

The performance of all ten models was comprehensively evaluated on the internal validation set from three perspectives:

Discrimination: Assessed by calculating the AUC with its 95% CI and plotting ROC curves.

Calibration: Assessed by plotting calibration curves to evaluate the agreement between predicted probabilities and observed frequencies.

Clinical utility was evaluated using decision curve analysis (DCA) to quantify the net benefit of the models across a range of threshold probabilities.

Additionally, we calculated several classification metrics, including accuracy, sensitivity (recall), specificity, positive predictive value (PPV or precision), negative predictive value (NPV), and the F1-score.

### Interpretability of the optimal model

To improve the interpretability of the model with the highest internal validation AUC [LR(L1/EN)], we used SHAP (SHapley Additive exPlanations) analysis. A global feature importance plot based on mean absolute SHAP values was generated to describe the relative contribution of each predictor to model output. A SHAP summary plot was further created to visualize the direction and magnitude of feature effects at the individual-patient level.

## Results

### Study population and baseline characteristics

In total, 485 consecutive adult patients who underwent intramedullary fixation for isolated tibial or femoral shaft fractures met the eligibility criteria and were included in the final complete-case analysis. Fracture union was achieved in 425/485 patients (87.6%), whereas 60/485 patients (12.4%) developed delayed union/nonunion at 6 months.

The distribution of baseline characteristics according to healing status is presented in Table 1. Individuals who developed delayed union/nonunion were significantly older than those who achieved union (48.5 ± 12.1 vs. 42.3 ± 13.5 years, *P* = 0.002). The proportion of male patients was comparable between groups (*P* = 0.651).

**Table 1:** Baseline characteristics of the study population stratified by fracture healing outcome

Characteristic	Overall (*n* = 485)	Healing Group (*n* = 425)	Delayed Union/Nonunion Group (*n* = 60)	*P*-value
Demographics				
Age (years)	43.1 ± 13.5	42.3 ± 13.5	48.5 ± 12.1	0.002
Sex (Male), n (%)	333 (68.7)	290 (68.2)	43 (71.7)	0.651
Comorbidities & Lifestyle				
Smoking, n (%)	150 (30.9)	121 (28.5)	29 (48.3)	0.003
Diabetes mellitus, n (%)	83 (17.1)	64 (15.1)	19 (31.7)	0.002
Fracture Characteristics				
Fracture location (Femur), n (%)	230 (47.4)	201 (47.3)	29 (48.3)	0.812
AO classification, n (%)				0.004
Type A	223 (46.0)	205 (48.2)	18 (30.0)	
Type B	125 (25.8)	110 (25.9)	15 (25.0)	
Type C	137 (28.2)	110 (25.9)	27 (45.0)	
Injury mechanism (High-energy), n (%)	373 (76.9)	325 (76.5)	48 (80.0)	0.589
Anthropometric & Biochemical Markers				
BMI (kg/m²)	24.8 ± 3.4	24.5 ± 3.2	26.8 ± 3.8	< 0.001
Waist circumference (cm)	86.0 ± 10.2	85.1 ± 9.8	92.4 ± 10.5	< 0.001
Triglycerides (mmol/L), median (IQR)	1.5 (1.1–2.1)	1.4 (1.0–1.9)	2.1 (1.5–2.9)	< 0.001
HDL-C (mmol/L)	1.23 ± 0.28	1.25 ± 0.28	1.08 ± 0.25	< 0.001
Albumin (g/L)	40.1 ± 4.2	40.2 ± 4.1	39.5 ± 4.5	0.215
VAI, median (IQR)	2.1 (1.4–3.1)	1.9 (1.3–2.8)	3.1 (2.2–4.5)	< 0.001

Data are presented as mean ± standard deviation *SD*, median (interquartile range *IQR*), or number (%). *P*-values were calculated using Student’s t-test or Mann–Whitney U test for continuous variables and the chi-square test or Fisher’s exact test for categorical variables. VAI is a unitless index

Significant *P*-values (*P* < 0.05) are highlighted in bold

Smoking and diabetes mellitus were more prevalent among patients with delayed union/nonunion. Specifically, smoking was observed in 48.3% of the delayed union/nonunion group compared with 28.5% of the union group (*P* = 0.003), and diabetes mellitus occurred in 31.7% versus 15.1%, respectively (*P* = 0.002).

Regarding fracture characteristics, the AO/OTA classification differed significantly between groups (*P* = 0.004). Type C fractures were more common in the delayed union/nonunion group (45.0%) than in the healing group (25.9%). No statistically significant differences were noted for fracture location or injury mechanism.

Metabolic and anthropometric parameters differed between groups. The median VAI in the overall cohort was 2.1 (IQR, 1.4–3.1), and was higher in patients with delayed union/nonunion than in those who achieved fracture union [3.1 (IQR, 2.2–4.5) vs. 1.9 (IQR, 1.3–2.8), *P* < 0.001]. Patients with delayed union/nonunion had higher BMI, WC, and TG levels and lower HDL-C concentrations (all *P* < 0.001), whereas serum albumin did not differ significantly between groups (*P* = 0.215).

### Association between VAI and risk of delayed union/nonunion

Logistic regression analyses evaluating the association between VAI and delayed union/nonunion are summarized in Table 2. When modeled continuously, higher VAI was associated with increased odds of delayed union/nonunion. In the crude model, each 1-unit increase in VAI was associated with an OR of 1.88 (95% CI 1.58–2.24; *P* < 0.001). The association remained significant after adjustment for age and sex (OR 1.75, 95% CI 1.45–2.11; *P* < 0.001) and after full multivariable adjustment (OR 1.61, 95% CI 1.31–1.98; *P* < 0.001). In quartile analyses, patients in Q3 and Q4 had higher odds of delayed union/nonunion than those in Q1 in the fully adjusted model (Q3: OR 3.62, 95% CI 1.51–8.67; Q4: OR 5.81, 95% CI 2.45–13.76; both *P* < 0.01). The trend across quartiles was significant in all models (*P* for trend < 0.001).

**Table 2:** Association between VAI and delayed union/nonunion in logistic regression models

Variable	Model 1 (Unadjusted)	Model 2 (Adjusted for Age, Sex)	Model 3 (Fully Adjusted*)
	OR (95% CI) *P*-value	OR (95% CI) *P*-value	OR (95% CI) *P*-value
VAI (continuous, per 1-unit increase)	1.88 (1.58–2.24) < 0.001	1.75 (1.45–2.11) < 0.001	1.61 (1.31–1.98) < 0.001
VAI (quartiles)			
Q1 (< 1.4)	1.00 (Reference)	1.00 (Reference)	1.00 (Reference)
Q2 (1.4–2.1)	2.10 (0.88–5.01) 0.091	2.01 (0.83–4.87) 0.118	1.95 (0.78–4.85) 0.153
Q3 (2.2–3.3)	4.15 (1.81–9.52) 0.001	3.89 (1.65–9.15) 0.002	3.62 (1.51–8.67) 0.004
Q4 (> 3.3)	7.21 (3.18–16.32) < 0.001	6.55 (2.81–15.26) <0.001	5.81 (2.45–13.76) < 0.001
P for trend	< 0.001	< 0.001	< 0.001

*Model 3 was fully adjusted for age, sex, smoking, diabetes mellitus, AO classification, and injury mechanism

Significant *P*-values (*P* < 0.05) are highlighted in bold

### Dose–response relationship between VAI and delayed union/nonunion

**Fig. 2:**
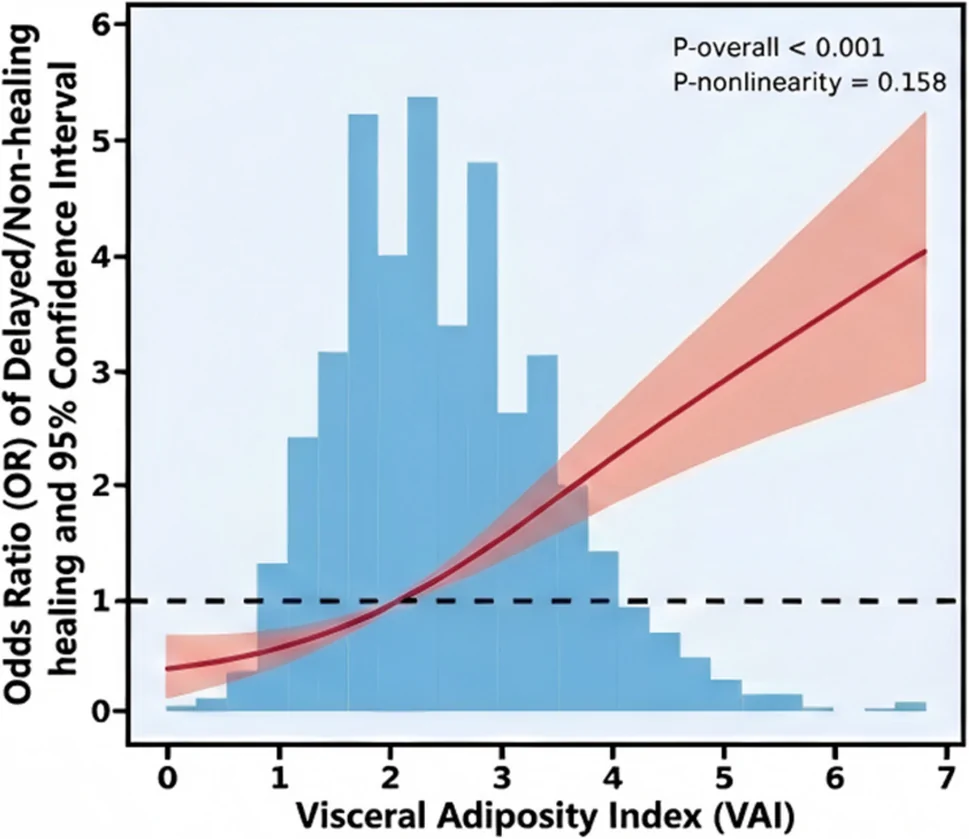
Dose-response relationship between the VAI and the risk of delayed union/nonunion of long bone fractures. The solid red line represents the adjusted OR, and the shaded red area represents the 95% CI, derived from a RCS regression model with four knots. The model was adjusted for age, sex, smoking, diabetes mellitus, AO classification, and injury mechanism. The reference value (OR = 1.0) was set at the median VAI of 2.1. The background histogram shows the distribution of VAI in the study population. The overall association was statistically significant (P for overall association < 0.001), whereas the test for nonlinearity was not significant (P for nonlinearity = 0.158), suggesting an approximately linear dose-response relationship

The functional form of the association between VAI and delayed union/nonunion was further explored using restricted cubic spline modeling (Fig. 2). After controlling for covariates, the overall relationship remained statistically significant (*P* < 0.001). No significant deviation from linearity was detected (P for nonlinearity = 0.158), indicating that risk increased in an approximately linear fashion across the VAI range.

### Subgroup analysis stratified by sex

**Fig. 3:**
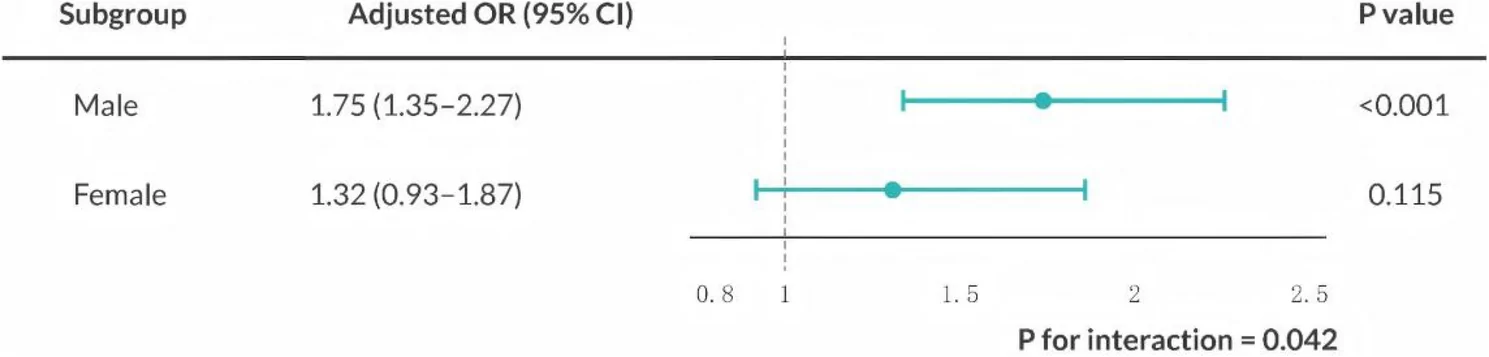
Subgroup analysis of the association between VAI and the risk of delayed union/nonunion, stratified by sex. The forest plot displays the adjusted ORs and 95% CIs for the association of a 1-unit increase in VAI with the outcome in male and female subgroups. The model was fully adjusted for age, smoking, diabetes mellitus, AO classification, and injury mechanism. A significant interaction between VAI and sex was observed (P for interaction = 0.042), suggesting that the association between VAI and delayed union/nonunion may differ by sex

Sex-specific analyses are illustrated in Fig. 3. In both male and female subgroups, higher VAI levels were associated with increased odds of delayed union/nonunion. A statistically significant interaction between VAI and sex was observed (P for interaction = 0.042), suggesting that the magnitude of association differed between men and women.

### Comparison of predictive performance between VAI and traditional obesity measures

**Fig. 4:**
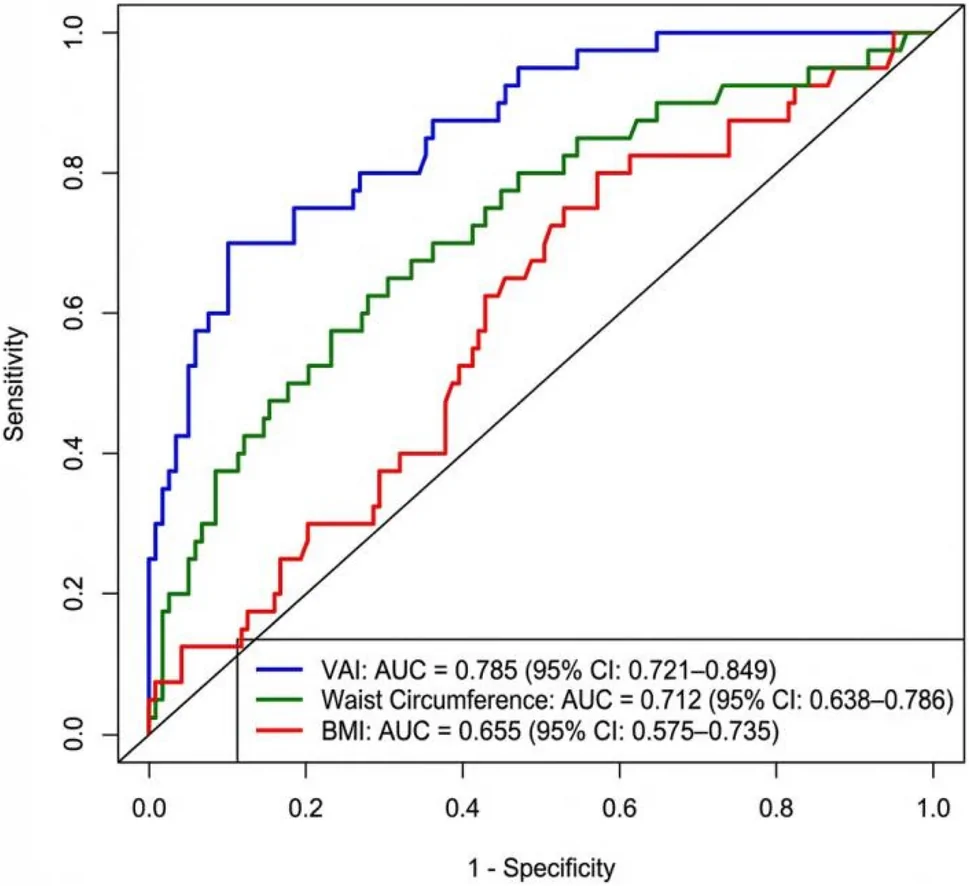
ROC curves comparing the discriminative performance of VAI, waist circumference, and BMI for delayed union/nonunion after tibial or femoral shaft fractures. The AUC for VAI was 0.785 (95% CI 0.721–0.849), which was higher than that for waist circumference (AUC 0.712, 95% CI 0.638–0.786; *P* = 0.031) and BMI (AUC 0.655, 95% CI 0.575–0.735; *P* = 0.008)

The discriminative capacity of VAI was compared with BMI and waist circumference using ROC analysis (Fig. 4). VAI yielded an AUC of 0.785 (95% CI 0.721–0.849), exceeding that of waist circumference (AUC 0.712; *P* = 0.031) and BMI (AUC 0.655; *P* = 0.008), indicating superior predictive performance.

### Predictive performance of machine learning models

**Fig. 5:**
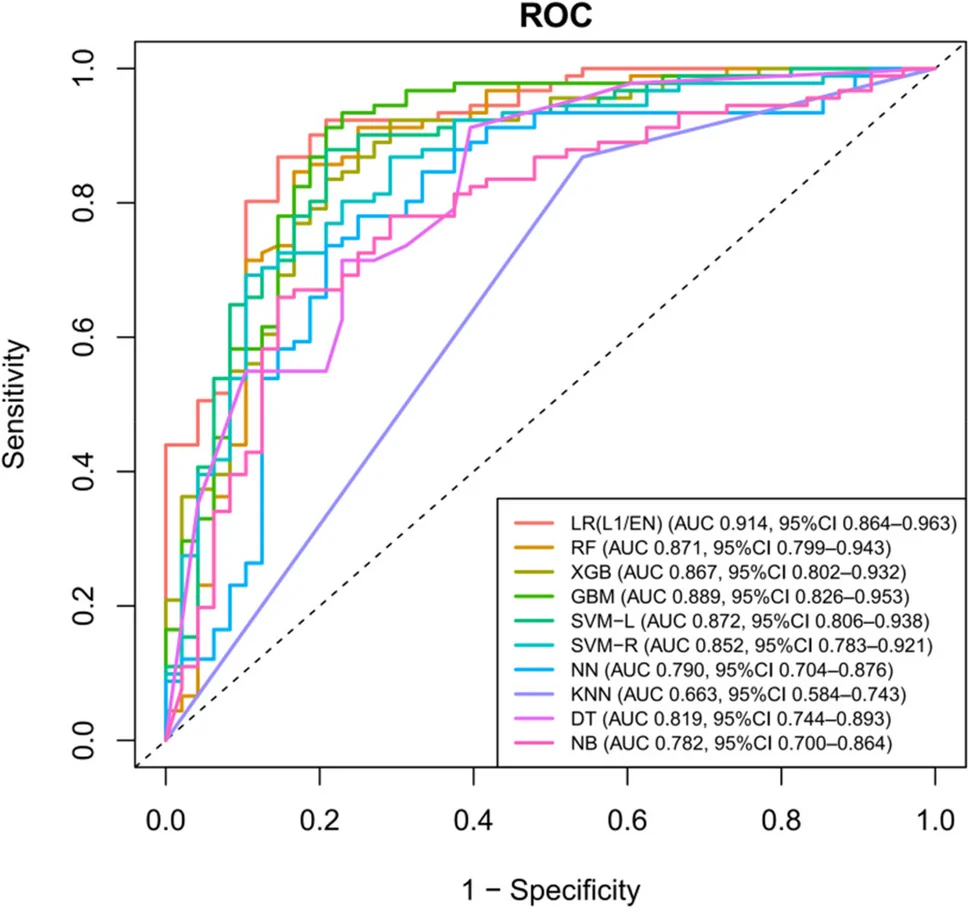
ROC curves of the ten machine learning models for predicting delayed union/nonunion in the validation set. The curves illustrate the discriminative performance of each model by plotting sensitivity against 1-specificity. In this internal validation analysis, the LR(L1/EN) model showed the highest AUC among the evaluated models

The performance of ten machine learning models was evaluated in the internal validation set (Table 3; Fig. 5). In the exploratory internal validation analysis, Elastic Net–regularized logistic regression showed the highest AUC among the evaluated models (AUC 0.914, 95% CI 0.864–0.963). This model also showed relatively balanced sensitivity, specificity, accuracy, and F1-score in the validation set. Given the single-center retrospective design and absence of external validation, these predictive performance estimates should be interpreted cautiously.

**Table 3:** Performance of exploratory machine learning models for delayed union/nonunion in the internal validation set

Model	AUC (95% CI)	Accuracy	Sensitivity (Recall)	Specificity	PPV (Precision)	NPV	F1-score
LR (L1/EN)	0.914 (0.864–0.963)	0.895	0.867	0.901	0.875	0.898	0.871
GBM	0.889 (0.826–0.953)	0.871	0.833	0.880	0.841	0.875	0.837
SVM-L	0.872 (0.806–0.938)	0.865	0.817	0.878	0.833	0.868	0.825
RF	0.871 (0.799–0.943)	0.862	0.825	0.872	0.825	0.872	0.825
XGB	0.867 (0.802–0.932)	0.858	0.808	0.871	0.819	0.864	0.813
SVM-R	0.852 (0.783–0.921)	0.840	0.792	0.853	0.801	0.847	0.796
DT	0.819 (0.744–0.893)	0.823	0.783	0.834	0.790	0.829	0.786
NN	0.790 (0.704–0.876)	0.805	0.750	0.819	0.763	0.809	0.756
NB	0.782 (0.700–0.864)	0.789	0.742	0.801	0.750	0.796	0.746
KNN	0.663 (0.584–0.743)	0.712	0.650	0.729	0.667	0.716	0.658

The best performance values for each metric are highlighted in bold

**Fig. 6:**
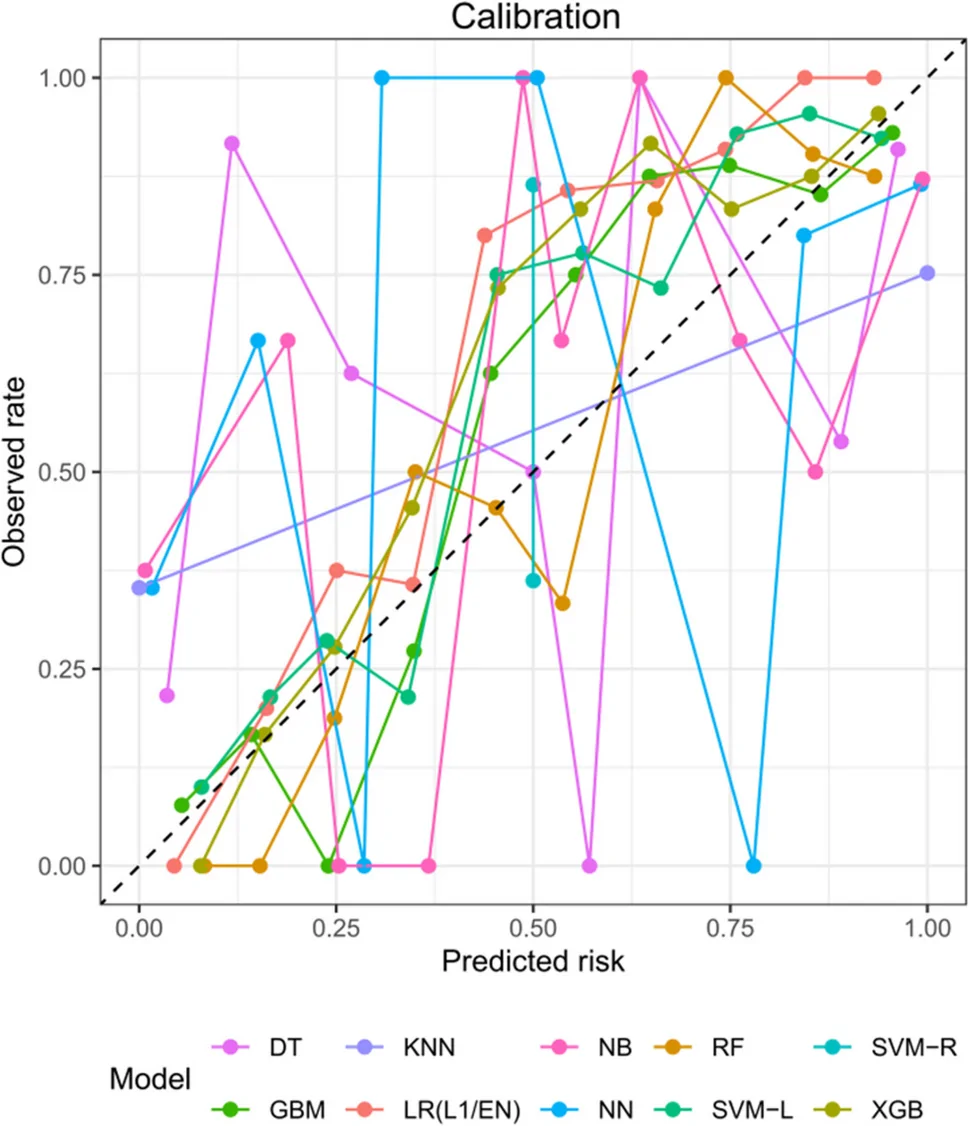
Calibration curves for the ten machine learning models in the validation set. The plot assesses the agreement between predicted probabilities of delayed union/nonunion and observed frequencies. The dashed diagonal line represents perfect calibration. The LR(L1/EN) model showed relatively close agreement with the ideal line in this internal validation set

**Fig. 7:**
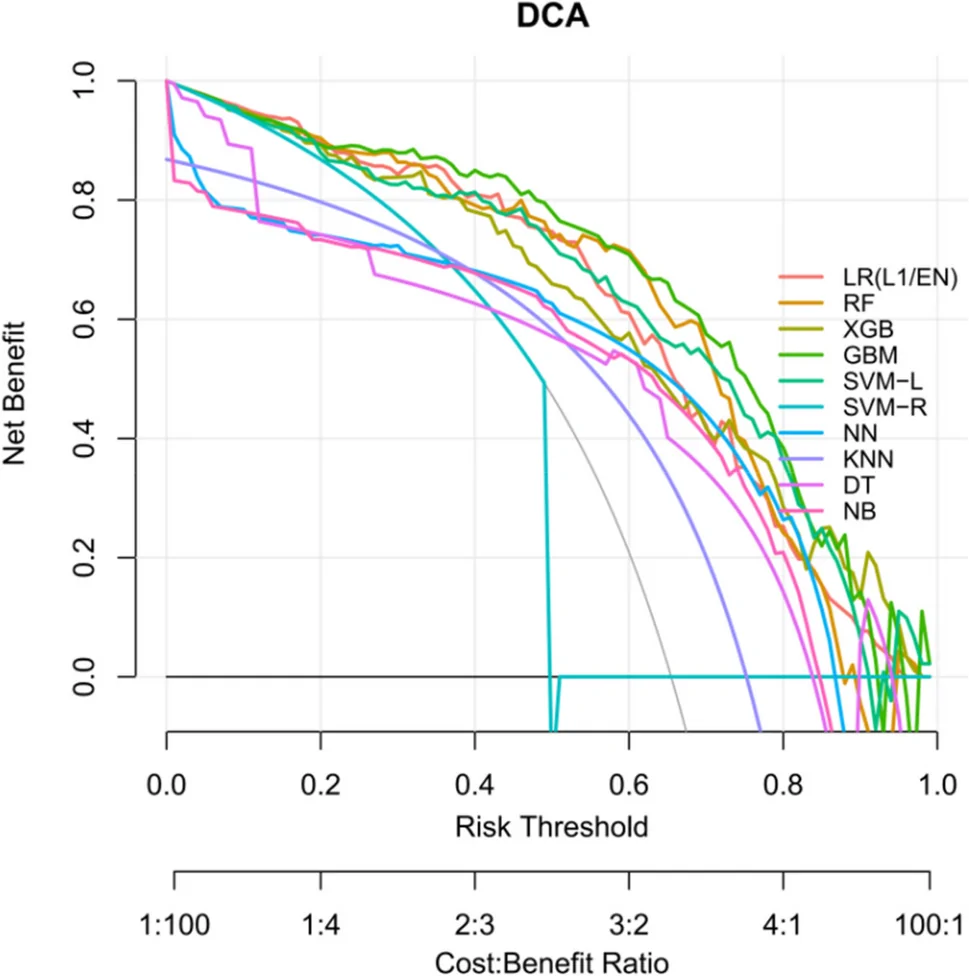
Decision curve analysis of the ten machine learning models in the validation set. The plot evaluates the net benefit of each model across a range of threshold probabilities. The horizontal black line represents the “treat none” strategy, and the gray line represents the “treat all” strategy. The LR(L1/EN) model showed higher net benefit across selected threshold ranges in this internal validation set

Calibration assessment showed relatively close agreement between predicted and observed probabilities for the LR(L1/EN) model (Fig. 6). Decision curve analysis suggested that this model had higher net benefit across selected threshold ranges in the internal validation set (Fig. 7).

### Model interpretability analysis based on SHapley Additive exPlanations

**Fig. 8:**
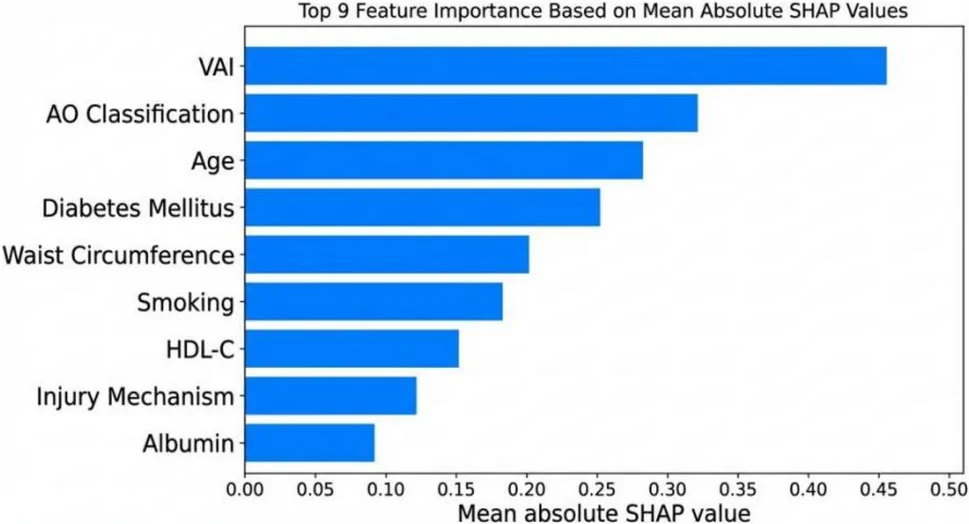
Global feature importance based on SHAP values. The plot ranks predictive features by their mean absolute SHAP values derived from the LR(L1/EN) model in the validation set. The x-axis indicates the mean absolute SHAP value, and the y-axis indicates the predictive features. Higher mean absolute SHAP values indicate greater average contribution to model output. VAI showed the highest mean absolute SHAP value among the included predictors

**Fig. 9:**
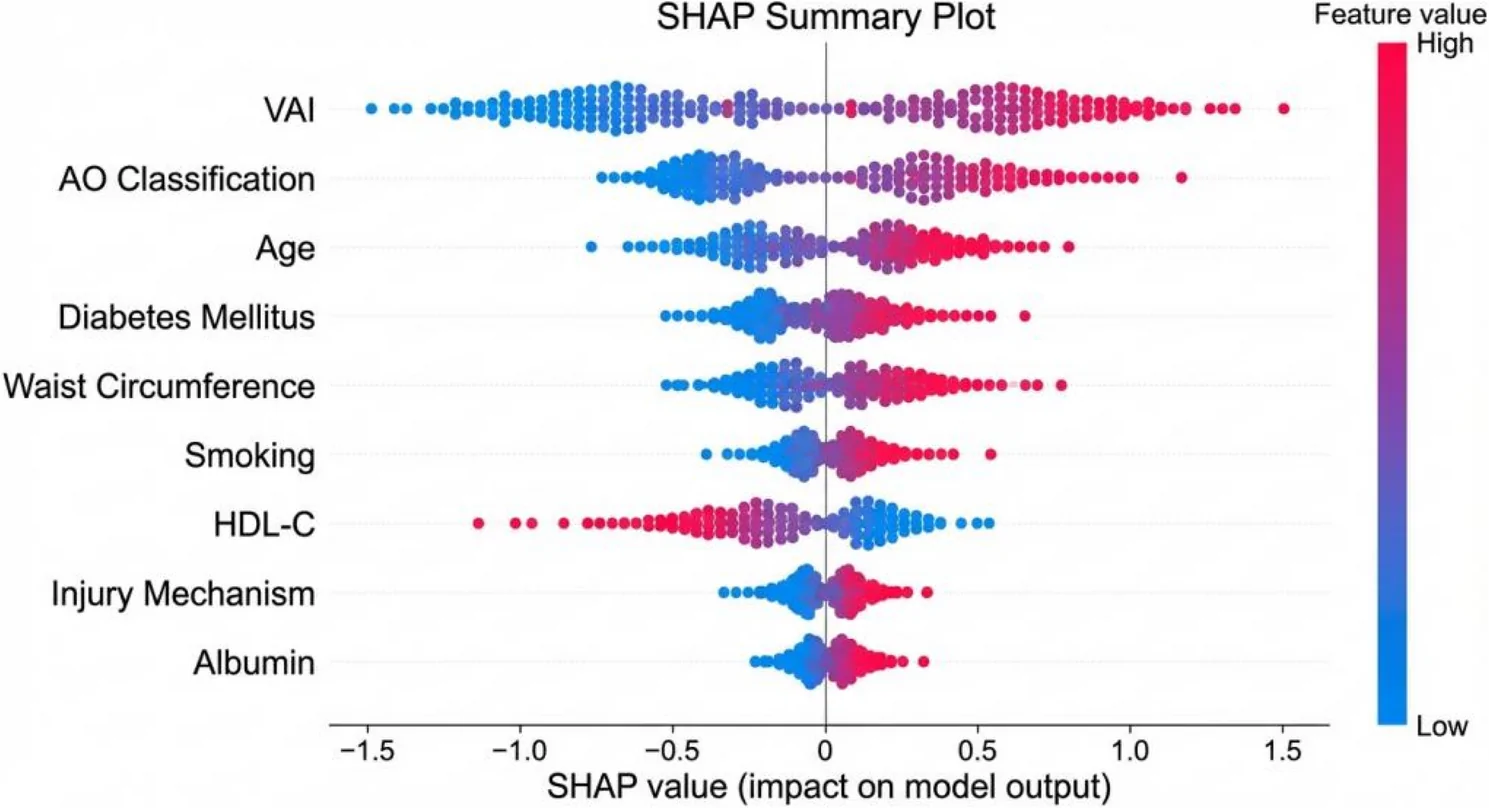
SHAP summary plot illustrating feature effects on model output. Each point on a feature row represents a single patient. The horizontal position indicates the SHAP value, and the color denotes the feature value. Higher VAI values, more complex AO classification, and older age were associated with higher predicted probabilities of delayed union/nonunion, whereas higher HDL-C levels were associated with lower predicted probabilities

SHAP analysis was applied to the LR(L1/EN) model to improve interpretability. Global importance rankings showed that VAI had the highest mean absolute SHAP value, followed by AO classification and age (Fig. 8). The SHAP summary plot suggested that higher VAI values, more complex fracture types, and older age contributed to higher predicted probabilities of delayed union/nonunion, whereas higher HDL-C levels were associated with lower predicted probabilities (Fig. 9).

## Discussion

In this retrospective cohort of 485 patients with long bone fractures, we systematically examined the association between VAI and postoperative delayed union/nonunion. Higher VAI was associated with delayed union/nonunion, and this association persisted after adjustment for age, smoking, diabetes mellitus, and fracture classification. Quartile analysis demonstrated a graded increase in risk across increasing VAI categories, and restricted cubic spline modeling indicated a predominantly linear association. In internal analyses, VAI showed higher discriminative performance than BMI and WC and was among the leading contributors in the exploratory machine learning model. Collectively, these findings suggest that visceral adiposity–related metabolic dysfunction may be associated with impaired fracture healing outcomes in this cohort.

The observed sex interaction should be interpreted cautiously. Sex-related differences in visceral fat distribution, lipid metabolism, hormone profiles, and adipokine-mediated inflammation may partly explain the different magnitude of association between VAI and delayed union/nonunion in men and women. In addition, VAI is calculated using sex-specific equations, which may capture metabolic dysfunction differently by sex. Given the exploratory nature of the subgroup analysis and the limited number of events, this finding should be considered hypothesis-generating and requires further validation.

Fracture healing is a complex and tightly regulated regenerative process involving inflammatory activation, angiogenesis, endochondral ossification, and subsequent bone remodeling [20–22]. Dysregulated or persistent inflammation has been recognized as a key contributor to impaired repair [23]. Chronic low-grade inflammatory states may interfere with osteoblast differentiation, enhance osteoclast activity, and disrupt vascular formation, thereby delaying bone regeneration [24]. Increasing evidence indicates that obesity-related inflammation and adipokine imbalance can modulate skeletal metabolism through interactions within the bone–immune axis [25, 26]. Mediators such as TNF-α, IL-6, and leptin participate in regulating osteoblast–osteoclast balance [27]. In this context, visceral adiposity-related dysfunction may be associated with an unfavorable systemic metabolic and inflammatory environment for fracture repair.

Unlike BMI, which primarily reflects overall body mass, VAI incorporates lipid parameters and may better capture functional alterations in adipose tissue. Previous studies have shown that VAI is associated with insulin resistance and metabolic syndrome, supporting its role as a surrogate marker of adipose tissue dysfunction and cardiometabolic risk [28–30]. The approximately linear relationship observed in our analysis suggests that metabolic dysregulation may be associated with a cumulative increase in delayed union/nonunion risk, rather than being confined to extreme obesity phenotypes.

Established risk factors for nonunion include smoking, diabetes mellitus, high-energy trauma, and complex fracture patterns [31–33]. Smoking may impair healing through vasoconstriction and reduced tissue oxygenation [34], whereas diabetes mellitus is associated with microvascular dysfunction and altered collagen deposition [35]. Although these factors remain clinically relevant, they represent isolated risk domains and may not fully reflect the broader metabolic milieu. In our multivariable models, VAI retained statistical significance after adjustment for these traditional predictors, indicating that it may provide incremental information beyond conventional risk stratification frameworks.

Most existing nonunion prediction tools are largely based on radiographic scoring systems or postoperative clinical variables [17–19], which may limit early risk identification. Incorporating metabolic indicators into prediction models may enable earlier stratification, potentially at the preoperative stage.

From a modeling standpoint, we developed exploratory machine learning models and evaluated their performance in an internal validation set. Therefore, the relatively high AUC of the LR(L1/EN) model should be interpreted as an internal performance estimate rather than evidence of external generalizability. Machine learning applications in orthopaedic trauma have expanded in recent years, particularly in postoperative complication prediction and imaging analysis [36, 37]. However, systematic reviews have highlighted methodological limitations in many published models, including small sample sizes, incomplete reporting, and limited external validation [38]. In the present study, reporting was informed by TRIPOD + AI guidance [35], and risk-of-bias considerations were informed by the PROBAST framework [39]. Clinical utility was assessed using decision curve analysis [40], and AUC comparisons were performed with the DeLong method [41]. These procedures were used to improve reporting transparency and methodological clarity.

SHAP analysis further improved model transparency and demonstrated that VAI was among the leading contributors to prediction. Explainable machine learning approaches are increasingly recognized as essential for clinical translation of predictive models.

From a clinical perspective, VAI can be calculated from routinely available anthropometric and lipid measurements at admission. In patients undergoing intramedullary fixation for tibial or femoral shaft fractures, elevated VAI may help identify individuals who require closer radiographic follow-up, early metabolic assessment, nutritional optimization, smoking cessation support, diabetes control, and individualized rehabilitation planning. However, VAI should be considered an adjunctive risk indicator rather than a standalone decision-making tool. Future multicenter prospective studies involving diverse populations are needed to validate the generalizability and clinical utility of VAI-based risk stratification.

Several limitations should be noted. First, this single-center retrospective study may be affected by selection bias and residual confounding. Some relevant factors, including vitamin D status, inflammatory markers, physical activity, anti-inflammatory drug use, rehabilitation adherence, and weight-bearing compliance, were unavailable. Second, admission lipid parameters may have been influenced by acute trauma-related stress; future studies with serial lipid measurements may provide more stable metabolic assessment. Third, potential predictor redundancy and multicollinearity should be considered because VAI is derived from BMI, WC, TG, and HDL-C. Finally, the machine learning models were exploratory and internally validated only, and this observational study cannot establish causality.

## Conclusion

In this single-center retrospective cohort of patients with tibial or femoral shaft fractures treated with intramedullary fixation, higher VAI was associated with an increased risk of delayed union/nonunion at 6 months. VAI showed better discrimination than BMI and waist circumference in internal analyses and may provide additional information for early risk stratification. However, these findings do not establish causality, and the prediction model requires external prospective validation before broader clinical application.

## Data Availability

The datasets used and/or analyzed during the current study are available from the corresponding author on reasonable request.
